# The Significant Impact of Narcan Reversals on Overdose Mortality in Peoria County, IL

**DOI:** 10.7759/cureus.29918

**Published:** 2022-10-04

**Authors:** Mohammad Mousbah AL-Tabbaa, Ehssan Bdiwi, Kathryn Endress, Khaled Altarras

**Affiliations:** 1 Internal Medicine, University of Illinois College of Medicine Peoria, Peoria, USA; 2 Internal Medicine, Kalamon University, Damascus, SYR; 3 Public Health, Peoria City/County Health Department, Peoria, USA; 4 Dentistry, Damascus University, Faculty of Dentistry, Damascus, SYR

**Keywords:** opioid, public health program development, public health, overdose, peoria, narcan

## Abstract

Objectives: Determine the impact of Narcan administrations in Peoria County, IL on the number of overdose deaths by testing the statistical significance of the association between monthly Narcan reversals and overdose mortality. As well as re-shedding the light on the opioid pandemic post-COVID.

Methods: We collected data on Narcan reversals from hospital emergency departments, Emergency Medical Services (EMS), the County Sheriff’s Office, local police departments, and other agencies that distributed and/or administered Narcan in Peoria County from January through December 2018. Data for the 2018 overdose mortality was collected through vital records at the Peoria City/County Health Department.

Results: Results from simple linear regression suggest that a significant proportion of the total variation in overdose mortality over 2018 was predicted by the Narcan reversals, F(1, 11) = 5.872, p< 0.05. Multiple R^2^ indicates that approximately 30.7% of the variation in overdose mortality was predicted by the Narcan reversals. If there were 0 Narcan reversals, there would be 8.362 overdose deaths per month.

Conclusions: Narcan is known to save lives in cases of opioid overdose, and the need for increased administration campaigns is warranted to further battle the opioid epidemic. As this study has proven, Narcan administration has the potential to significantly decrease overdose mortality.

## Introduction

According to the CDC, “91,799 drug overdose deaths occurred in the United States in 2020”, opiates accounted for 82.3% of those deaths, with a 28.3% increase in overdose death in the state of Illinois [1-. In Peoria County, the total years of potential life lost due to overdoses in 2018 were 1,300 with an incidence rate of 24.6 per 100,000, affecting mainly African Americans and residents of lower-income zip codes. The CDC started an initiative in 2014 that allows laypersons to administer Narcan [2]. Illinois has joined the initiative with a standing order that allows pharmacists and Narcan training programs to distribute Narcan without a prescription [3]. Despite the increase in Narcan prescriptions, the overall quantity is low in comparison to high-dose opioid prescriptions. According to the CDC morbidity and mortality weekly report, “in 2018, one naloxone prescription was dispensed for every 69 high-dose opioid prescriptions” [4]. With the COVID pandemic affecting all aspects of public health services, we hope our study can shed the light again on the opioid epidemic. This article was previously presented at the American Society of Addiction Medicine (ASAM) annual meeting on April 20, 2020.

## Materials and methods

A retrospective study that investigated the association between Narcan administrations and overdose mortality in Peoria County, IL in 2018 was conducted. Data for Narcan administrations was collected from hospital emergency departments, Emergency Medical Services (EMS), the County Sheriff’s Office, local police departments, and other agencies that distributed and/or administered Narcan in Peoria from January through December of 2018. These agencies reported their Narcan administration to the Narcan advisory committee in Peoria City/County Health Department on a monthly basis. Narcan administrations for non-Peoria county residents were excluded as well as administrations with no reported date. Data for the 2018 overdose mortality was collected through vital records at the Peoria City/County Health Department. SPSS software (IBM Corp, Armonk, New York, USA) was used for statistical analysis.

## Results

**Figure 1:**
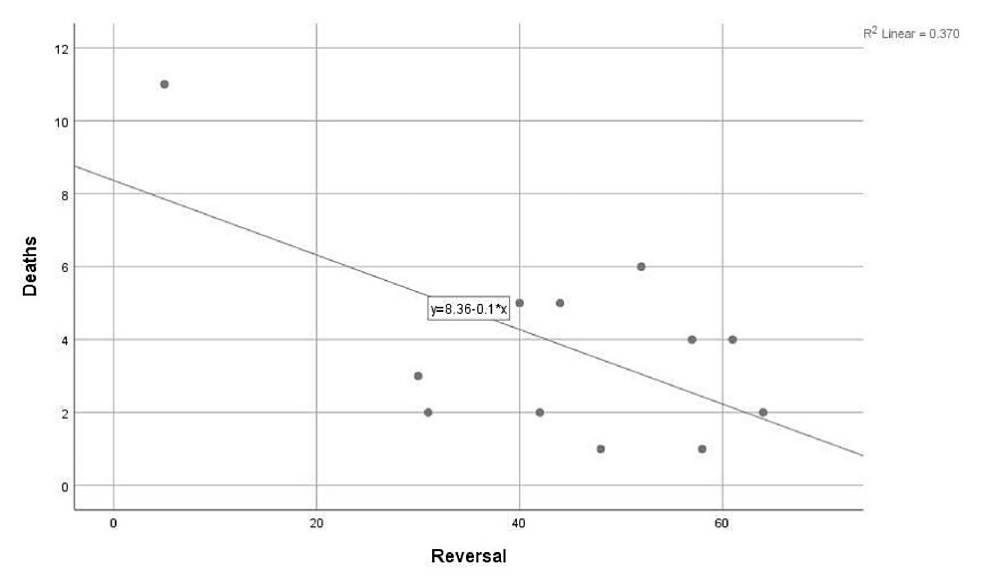
Scatter plot of Narcan reversals and overdose mortality in 2018.

A simple linear regression was performed to determine if Narcan reversals could predict overdose mortality in Peoria County in 2018. The scatterplot of the monthly Narcan reversals and monthly overdose mortality indicates that the assumption of linearity is reasonable. As monthly Narcan reversals increase, monthly overdose mortality decreases (Figure 1).

The results from Table 1 suggest that a significant proportion of the total variation in overdose mortality in 2018 was predicted by the Narcan reversals, F(1, 11) = 5.872, p < 0.05. Multiple R squared indicates that approximately 30.7% of the variation in overdose mortality was predicted by the Narcan reversals. If there were 0 Narcan reversals, there would be 8.362 overdose deaths per month in Peoria County.

**Table 1:** Summary of simple linear regression analysis for the variable predicting overdose mortality.

	Overdose Mortality		
	B_0_	SE B	r
Narcan reversals	8.362	1.985	-0.608*
R^2^		0.307	
F		5.872*	

The results from the analysis above highlighted the statistical significance of Narcan distribution in areas of high risk for opiate overdose to lower drug overdose mortality in Peoria County, IL.

## Discussion

A literature search was performed using the University of Illinois in Springfield Brookens Library database and PubMed using the terms “Naloxone Significance,” “Narcan mortality,” and “Covid impact on public health”. Limited studies were found that studied the significance of the Narcan administrations in decreasing overdose mortality as well as for the effects of COVID on public health. One study conducted in Australia evaluated the value of take-home naloxone programs in decreasing heroin fatalities between 1/2012 and 12/2013. They found that there was a slight reduction in mortality [5]. Another study done in Massachusetts evaluated post-Narcan administration one-year mortality for patients who received at least one Narcan administration by EMS between 7/1/13 and 12/31/15. They found that “Death records indicated that 6.5% (n=787) died the same day as the documented naloxone administration, 9.3% (n=1,132) died within one year and 84.3% (n=10,273) were alive at one year” [6]. A systematic review done in Boston, MA evaluated the effectiveness of take-home Narcan in decreasing overdose mortality among people with opioid use disorder. They found that “there is overwhelming support of take-home naloxone programs being effective in preventing fatal opioid overdoses” [7]. A study done in 2017 reviewed the use of Narcan in Opioid overdose prevention [8]. That study raised an important question about who should prescribers be worried about “risk compensation”, meaning the risk of doing a high dose of opiates that will be compensated by the use of Narcan. Authors reviewed the Overdose Education and Naloxone Distribution (OEND) and its effectiveness when implicated before distributing Narcan in lowering opiate overdose mortality [9]. One study in Boston, MA compared the use of OEND in high-risk communities and a statistical significant decrease in opiate overdose was observed [10]. Another study in Scotland, UK studied the effectiveness of Scotland’s national naloxone program in a high-risk population and found that “Scotland’s National Naloxone Programme was associated with a 36% reduction in the proportion of opioid‐related deaths that occurred in the 4 weeks following release from prison” [11-12]. These studies have evaluated the effectiveness of Narcan in decreasing mortality in people who were identified as people with opioid use disorder only or labeled as high risk for opiate use. Whereas our study investigated the effectiveness of Narcan administrations in decreasing overdose mortality in the entire population, and it proved the statistical significance of increased Narcan administrations in decreasing overdose mortality.

Limitations of our study include that there was no report of drug type for each Narcan administration and a limited number of bystander-reported administrations.

Due to the urgency of the COVID-19 pandemic, it affected most public health services and halted most studies in other fields of public health such as the opiate epidemic as most studies had to be directed toward curtailing the pandemic [13]. A cross-sectional study done in the USA was able to highlight the effect of COVID-19 on other public health services, they found that “The provision of many essential public health functions and tasks have been limited or eliminated while the US public health workforce responds to the COVID-19 pandemic” [14]. Another review article found that COVID resulted in “worsening crisis in the control of sexually transmitted disease while at the same time have mitigated other public health problems such as flu, respiratory syncytial virus, and norovirus disease outbreaks.” As the public health system is recovering from the effects of the COVID-19 pandemic, it is imperative to re-shift the focus toward other public health issues such as the opiate crisis. It was the intention of publishing right now to re-open the discussion about the opiate crisis.

## Conclusions

This study has proven the statistical significance of Narcan administrations in decreasing overdose mortality in Peoria County reinforcing the need for increased access to Narcan to be a public health priority. Still, these campaigns are not enough to curtail the opioid crises. It’s recommended that future studies evaluate medication-assisted treatment programs and other programs’ effectiveness in decreasing the opioid epidemic burden. With the public health system starting to recover from COVID-19, it’s imperative to resume previous efforts in studying the opioid crisis, more studies are still needed to evaluate the current efforts in curtailing the opiate crisis as well as evaluating new and innovative methods.
